# Supplementary material for: TMEM16A in smooth muscle cells acts as a pacemaker channel in the internal anal sphincter
Source: Commun Biol. 2024 Feb 5;7:151. doi: 10.1038/s42003-024-05850-1 (PMC10844222; doi:10.1038/s42003-024-05850-1)
Supplement: Supplementary file 2 — Supplementary Information [file 42003_2024_5850_MOESM2_ESM.docx]

**TMEM16A in smooth muscle cells acts as a pacemaker channel in the internal anal sphincter**

Ping Lu^1^, Lawrence M Lifshitz^2^, Karl Bellve^2^, and Ronghua ZhuGe^1, Φ^

^1^Department of Microbiology and Physiological Systems, University of Massachusetts Chan Medical School, Worcester, MA, USA

^2^Program in Molecular Medicine, University of Massachusetts Chan Medical School, Worcester, MA, USA

### ^Φ^Corresponding author: Ronghua.zhuge@umassmed.edu

**Additional Information**

**The following Supplementary Material is available for this paper:**

1. Supplementary Figures 1-7 (available in this file)
2. Supplementary Movie 1 (available as an AVI file and a Word file for the caption)
3. Supplementary Data (available as an MS Excel file)


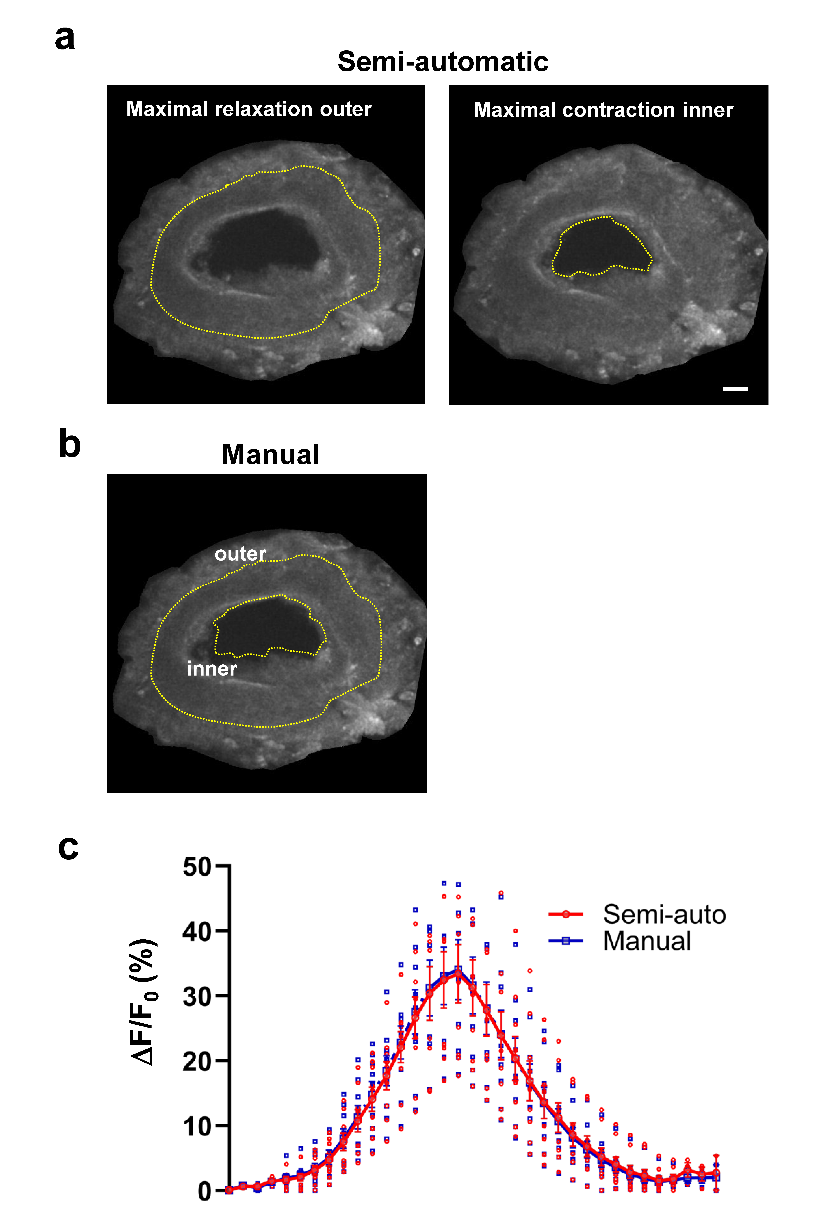


**Supplementary Fig. 1. Quantification of Ca^2+^ signals in the circular smooth muscle of IAS slices.** (a) Illustration of "semi-automatic" measurements of Ca^2+^ fluorescence during rhythmic contractions of the circular smooth muscle in IAS slices. We first manually determined the outer boundary (shown on the left) of the circular smooth muscle in the frame acquired at the time of maximal relaxation or minimal contraction, and the inner boundary (shown on the right) in the frame acquired at the time of maximal contraction. These two boundaries were then applied to all subsequent frames to extract the Ca^2+^ fluorescence data between them for subsequent analyses. Scale bar = 200 µm. (b) Illustration of manual measurements of Ca^2+^ fluorescence in the circular smooth muscle of IAS slices. Inner and outer boundaries were manually outlined in each frame. Fluorescence within these boundaries was then measured and calculated. Shown are two outlines from a single frame taken during a Ca^2+^ wave. (c) No significant difference in the dynamics or amplitude of Ca^2+^ signals was detected when measured by these two methods (n=9 events, p=0.99 by paired two-tailed Student’s t-test).


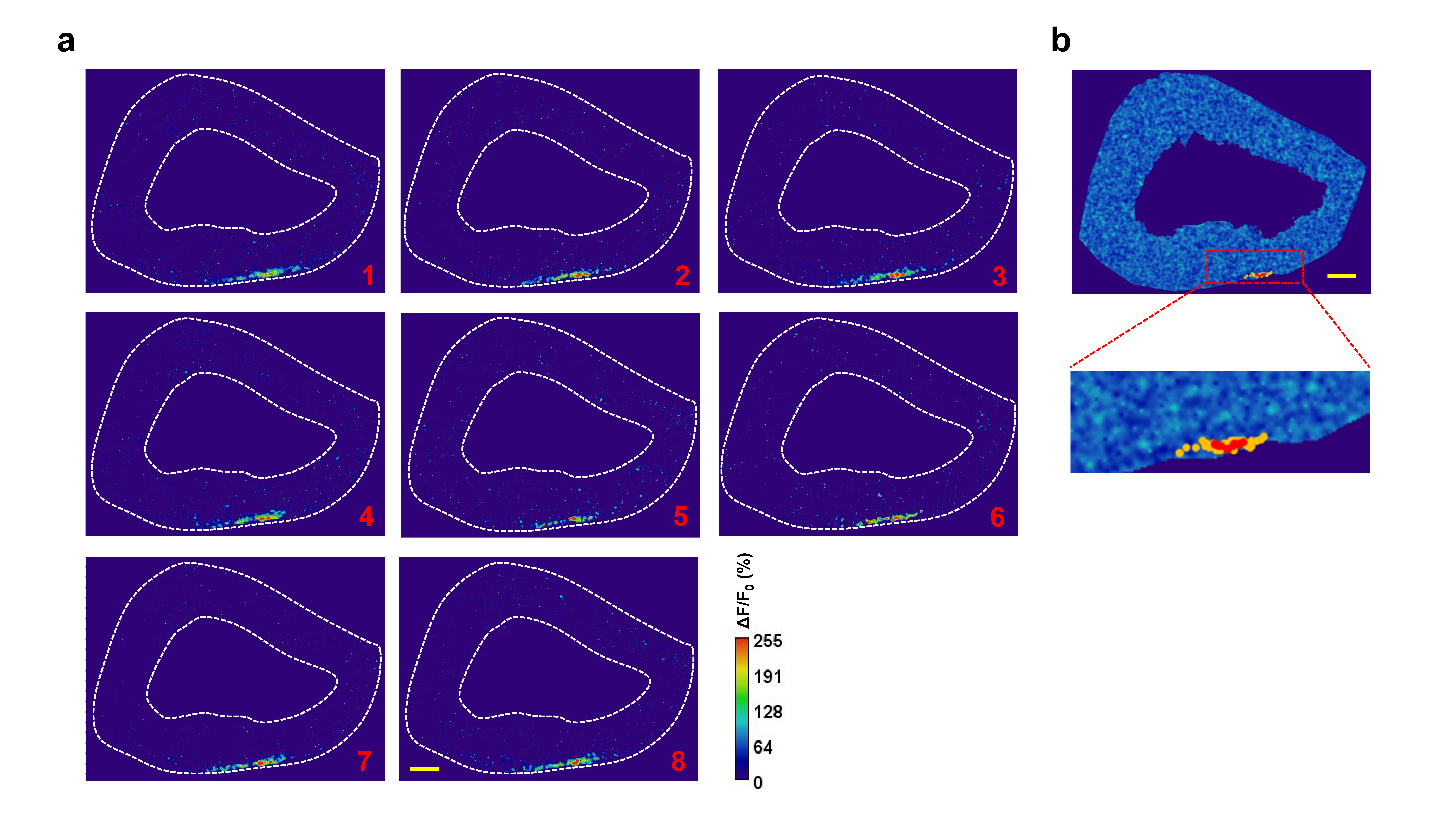


**Supplementary Fig. 2. Ca^2+^ waves originate at fixed locations within IAS slices.** (a) The images depict the initiation frames of eight Ca^2+^ waves in the same slice, as shown in Fig. 1d. (b) The initiation locations of Ca^2+^ waves within the slice are shown, as indicated in panel a. These locations are marked by filled dots and represent the points with the highest fluorescence values in the initiation frames, captured during a 2.5-min recording. An inset provides a magnified view of the location map, color-coded as follows: red indicates two repeats, and yellow indicates a single event. Scale bar = 200 μm.

**
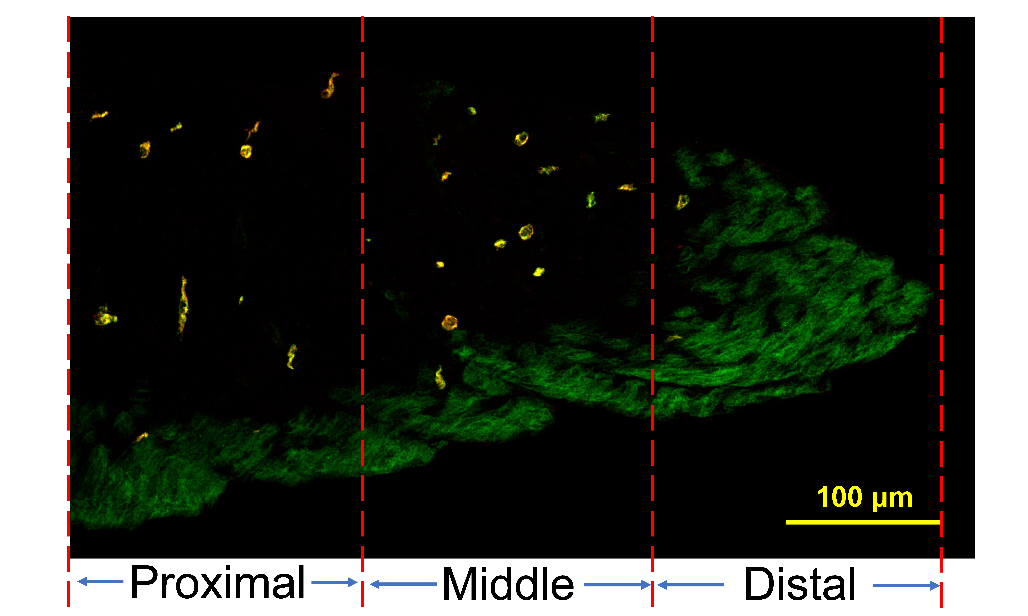
**

**Supplementary Fig. 3. Zonal delineation for quantification of TMEM16A expression in the IAS.** This figure illustrates the methodology for defining three predefined regions of equal longitudinal length: proximal, middle, and distal, for the quantification of TMEM16A expression. The image used for this illustration is identical to the one shown in the upper row, far right, of Fig. 5a.


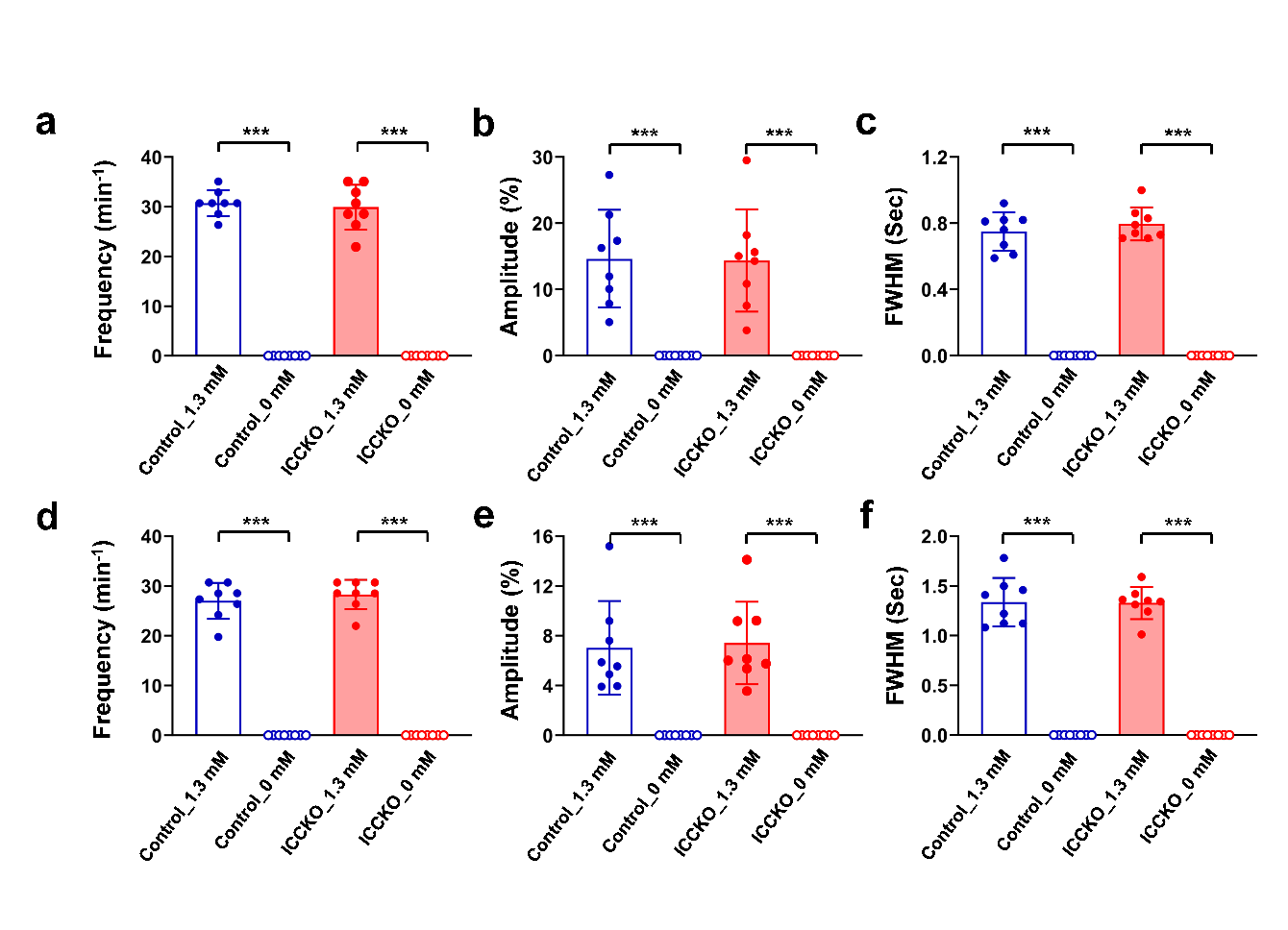


**Supplementary Fig. 4. Dependence of Ca^2+^ waves and rhythmic contractions on extracellular Ca^2+^ in IAS slices from isogenic control mice and TMEM16A^ICCKO^ mice.**  (a-c) Summarized results illustrating the effect of zero extracellular Ca^2+^ on the frequency, amplitude, and full width at half maximum (FWHM) of Ca^2+^ waves (i.e. ΔF/F_0_(%)) in IAS slices from both control (n = 8) and TMEM16A^ICCKO^ (ICCKO) mice (n = 8). (d-f) Summarized results showing the effects of zero extracellular Ca^2+^ on the frequency, amplitude, and FWHM of rhythmic contractions (i.e. changes in lumen area) in IAS slices from control (n = 8) and TMEM16A^ICCKO^ mice (n = 8). ***p<0.001, as determined by paired two-tailed Student’s t-test.

**
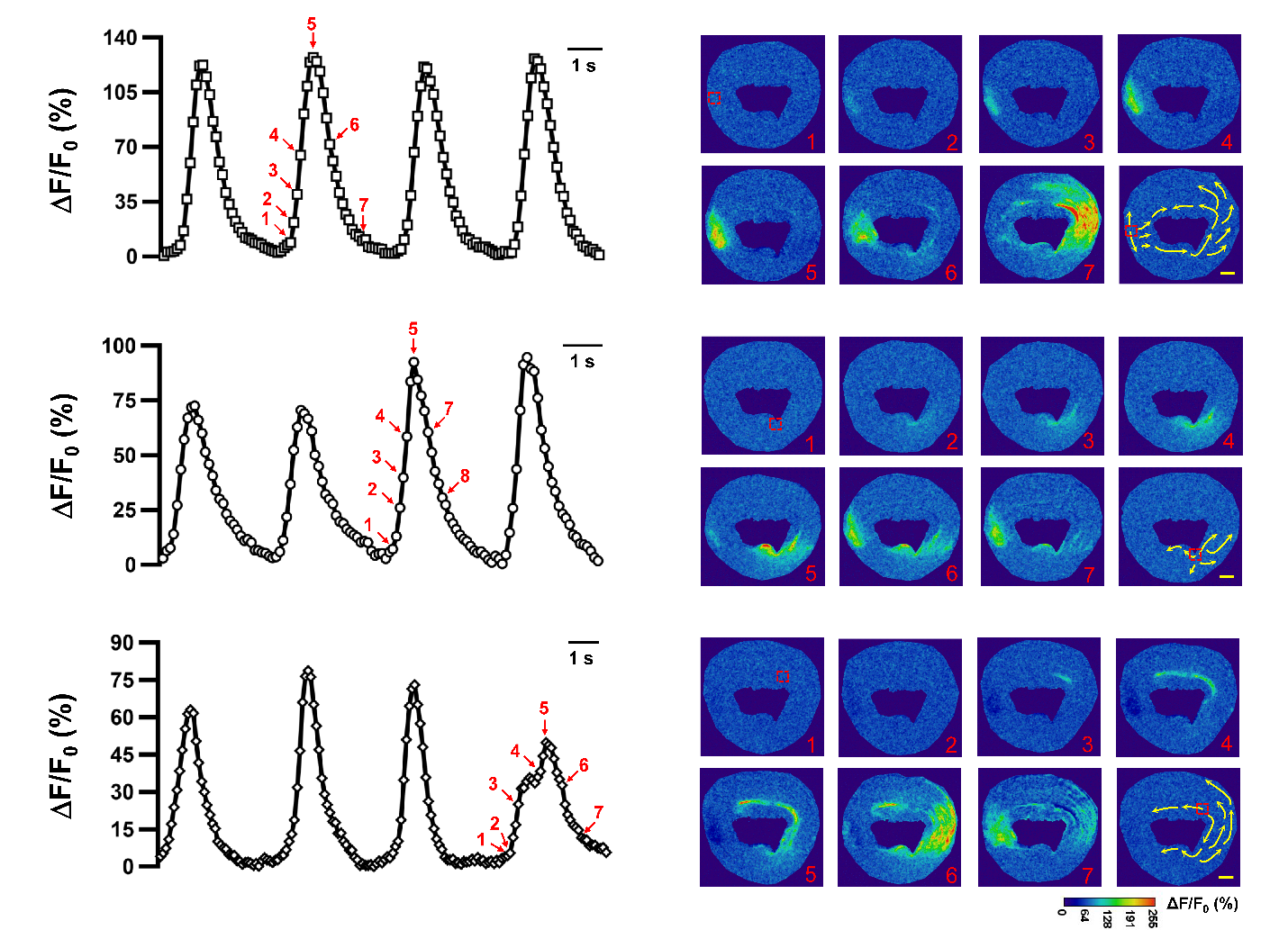
**

**Supplementary Fig. 5. Multiple pacemakers in IAS slices from TMEM16A^ICCKO^ mice.** This figure shows that IAS slices from TMEM16A^ICCKO^ mice have multiple pacemakers. Three representative Ca^2+^ waves are shown to originate consistently from these fixed points within an IAS slice from a TMEM16A^ICCKO^ mouse.

*Traces:* Time courses for three different Ca^2+^ waves are shown, each originating from a specific area delineated by a red square dotted box (10 × 10 pixels) in Image 1 on the right.

*Images:* The spatiotemporal evolution of a single Ca^2+^ wave is depicted. Image numbers correspond to those labeled near the adjacent trace. Arrows in the last image of each event highlight the primary propagation paths of each Ca^2+^ wave from its respective pacemaker.

**Note:** The behavior of Ca^2+^ waves in IAS slices from TMEM16A^ICCKO^ mice closely mirror that observed in control slices (see Fig. 2). Specifically, (1) Ca^2+^ waves propagated either parallel or perpendicular to the circular smooth muscle cells (SMCs), and (2) Ca^2+^ waves originating from different pacemakers could follow the same path and subsequently collide. Scale bar = 200 μm.

**
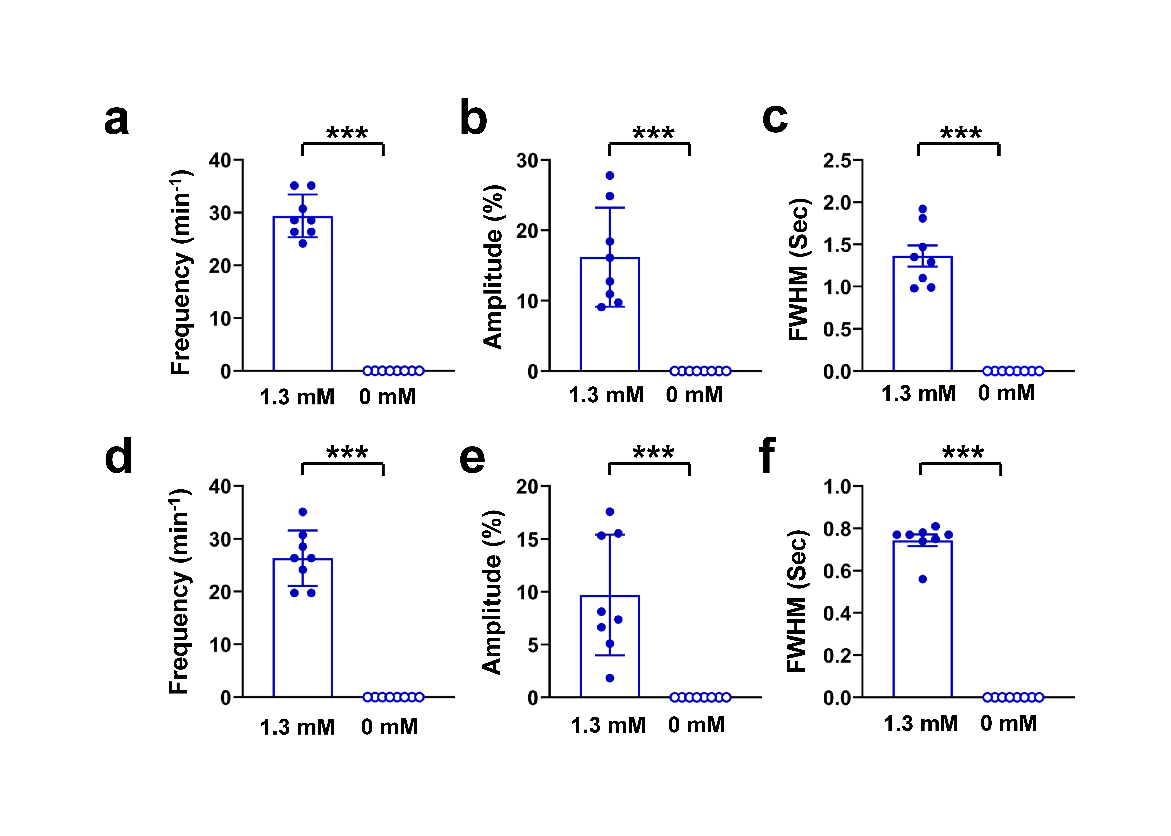
**

**Supplementary Fig. 6. Dependence of Ca^2+^ waves and rhythmic contractions on extracellular Ca^2+^ in IAS slices from isogenic control mice for TMEM16A^SMKO^ mice.** (a-c) Ca^2+^ Waves: Summarized results show the effect of zero extracellular Ca^2+^ on the frequency, amplitude, and full width at half-maximum (FWHM) of Ca^2+^ waves (i.e., ΔF/F_0_(%)). (d-f) Rhythmic contractions: Summarized results illustrate the effect of zero extracellular Ca^2+^ on the frequency, amplitude, and FWHM of rhythmic contractions. n = 8; ^***^p<0.001, determined by paired two-tailed Student’s t-test.


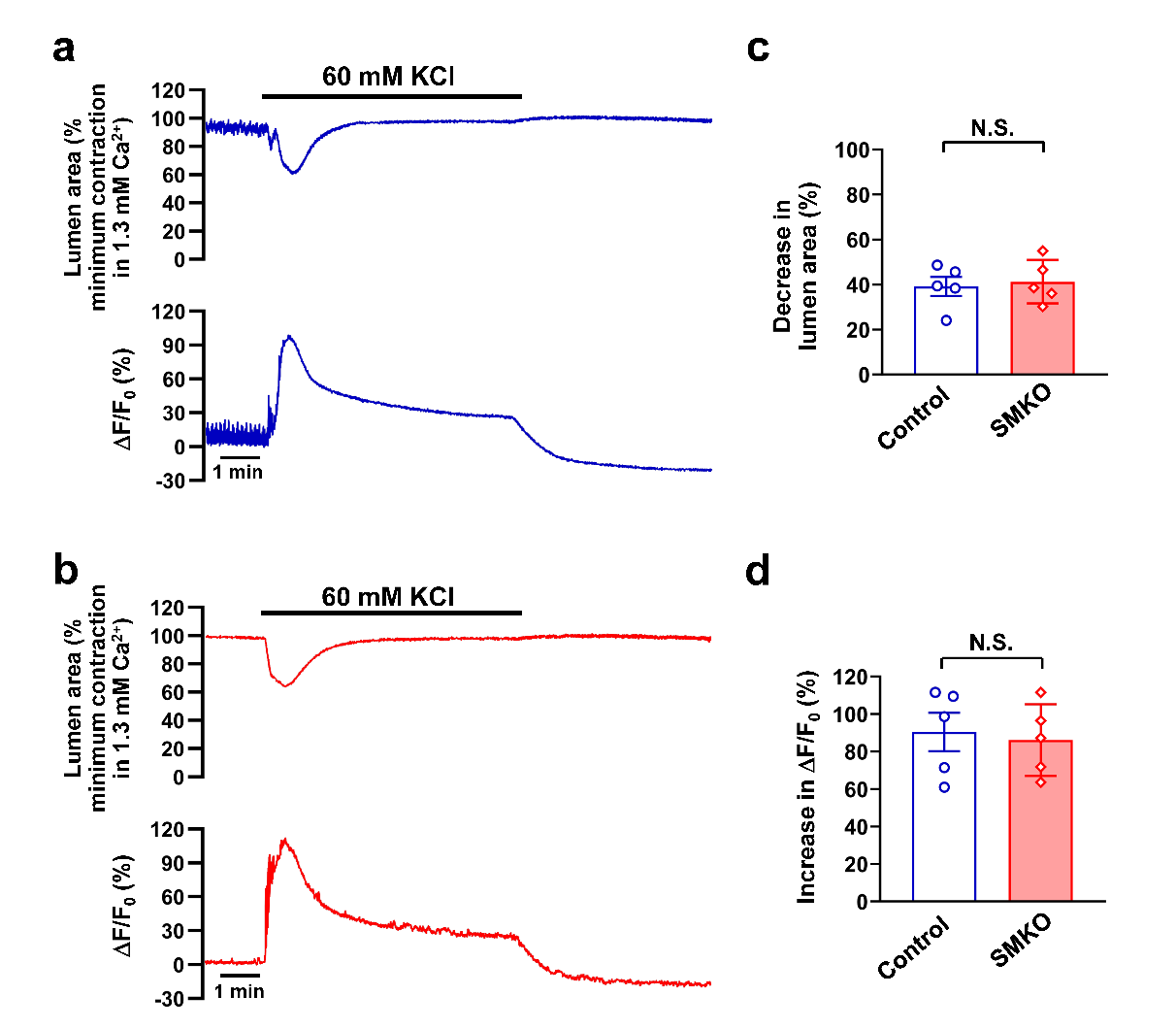


**Supplementary Fig. 7. High KCl induces similar increases in calcium and contractions in IAS slices from both TMEM16A^SMKO^ and isogenic control mice.** (a) Representative contraction (upper trace) and calcium signal (lower trace) recordings from an isogenic control IAS slice. (b) Representative contraction (upper trace) and calcium signal (lower trace) recordings from a TMEM16A^SMKO^ IAS slice. (c-d) Summarized results for calcium signals and contractions in IAS slices from both control (n = 5) and TMEM16A^SMKO^ mice (n = 5). N.S. indicates no statistical significance, as determined by unpaired two-tailed Student’s t-test.
