# Supplementary material for: TMEM16A in smooth muscle cells acts as a pacemaker channel in the internal anal sphincter
Source: Commun Biol. 2024 Feb 5;7:151. doi: 10.1038/s42003-024-05850-1 (PMC10844222; doi:10.1038/s42003-024-05850-1)
Supplement: Supplementary file 3 — Description of Additional Supplementary Files [file 42003_2024_5850_MOESM3_ESM.pdf]

Description of Additional Supplementary Files

File name: Supplementary Data 1
Description: This Excel file is the source data containing all the data presented in both the main and supplementary figures.

File name: Supplementary Movie 1
Description: Calcium waves originate from three pacemakers in the IAS slice, as shown in Fig. 2. Image acquisition speed: 11 Hz, and video playback speed: 33 Hz.
