# Supplementary material for: TMEM16A in smooth muscle cells acts as a pacemaker channel in the internal anal sphincter
Source: Commun Biol. 2024 Feb 5;7:151. doi: 10.1038/s42003-024-05850-1 (PMC10844222; doi:10.1038/s42003-024-05850-1)
Supplement: Supplementary file 6 — Reporting Summary [file 42003_2024_5850_MOESM6_ESM.pdf]

nature portfolio

Corresponding author(s): Ronghua ZhuGe

Last updated by author(s): Dec 26, 2023

Reporting Summary

Nature Portfolio wishes to improve the reproducibility of the work that we publish. This form provides structure for consistency and transparency in reporting. For further information on Nature Portfolio policies, see our Editorial Policies and the Editorial Policy Checklist.

Statistics

For all statistical analyses, confirm that the following items are present in the figure legend, table legend, main text, or Methods section.

n/a	Confirmed	
☐	☒	The exact sample size (*n*) for each experimental group/condition, given as a discrete number and unit of measurement
☐	☒	A statement on whether measurements were taken from distinct samples or whether the same sample was measured repeatedly
☐	☒	The statistical test(s) used AND whether they are one- or two-sided<br>*Only common tests should be described solely by name; describe more complex techniques in the Methods section.*
☒	☐	A description of all covariates tested
☒	☐	A description of any assumptions or corrections, such as tests of normality and adjustment for multiple comparisons
☐	☒	A full description of the statistical parameters including central tendency (e.g. means) or other basic estimates (e.g. regression coefficient) AND variation (e.g. standard deviation) or associated estimates of uncertainty (e.g. confidence intervals)
☐	☒	For null hypothesis testing, the test statistic (e.g. *F*, *t*, *r*) with confidence intervals, effect sizes, degrees of freedom and *P* value noted<br>*Give P values as exact values whenever suitable.*
☒	☐	For Bayesian analysis, information on the choice of priors and Markov chain Monte Carlo settings
☒	☐	For hierarchical and complex designs, identification of the appropriate level for tests and full reporting of outcomes
☒	☐	Estimates of effect sizes (e.g. Cohen's *d*, Pearson's *r*), indicating how they were calculated

Our web collection on statistics for biologists contains articles on many of the points above.

Software and code

Policy information about availability of computer code

Data collection	The software used for calcium signal image acquisition is µManager, which is an open source project hosted on GitHub.<br>The immunostaining images were acquired by Leica TCS SP8 confocal microscope (Leica Microsystems Inc., Wetzlar, Germany).<br>The isometric force were measured by a myograph system (610-M, Danish Myo Technology, Aarhus, Denmark).
Data analysis	The software employed for contraction, calcium analysis, and immunostaining signals co-localization analysis is ImageJ (http://imagesj.nih.gov).<br>The frequency and FWHM were analyzed by OriginPro 2022.

For manuscripts utilizing custom algorithms or software that are central to the research but not yet described in published literature, software must be made available to editors and reviewers. We strongly encourage code deposition in a community repository (e.g. GitHub). See the Nature Portfolio guidelines for submitting code & software for further information.

Data

Policy information about availability of data

All manuscripts must include a data availability statement. This statement should provide the following information, where applicable:

- Accession codes, unique identifiers, or web links for publicly available datasets
- A description of any restrictions on data availability
- For clinical datasets or third party data, please ensure that the statement adheres to our policy

The data that support the findings of this study are available from the corresponding author upon reasonable request.

Human research participants

Policy information about studies involving human research participants and Sex and Gender in Research.

Reporting on sex and gender	Not applicable.
Population characteristics	Not applicable.
Recruitment	Not applicable.
Ethics oversight	Not applicable.

Note that full information on the approval of the study protocol must also be provided in the manuscript.

Field-specific reporting

Please select the one below that is the best fit for your research. If you are not sure, read the appropriate sections before making your selection.

☒ Life sciences ☐ Behavioural & social sciences ☐ Ecological, evolutionary & environmental sciences

For a reference copy of the document with all sections, see nature.com/documents/nr-reporting-summary-flat.pdf

Life sciences study design

All studies must disclose on these points even when the disclosure is negative.

Sample size	For each experiment, the sample size is at least 5 (5≤n≤21), which was sufficient to reach statistical significance in our study.
Data exclusions	Not applicable.
Replication	We repeated each treatment for at least 5 tissue samples from 5 mice.
Randomization	Not applicable.
Blinding	Not applicable.

Reporting for specific materials, systems and methods

We require information from authors about some types of materials, experimental systems and methods used in many studies. Here, indicate whether each material, system or method listed is relevant to your study. If you are not sure if a list item applies to your research, read the appropriate section before selecting a response.

Materials & experimental systems

n/a	Involved in the study	
☐	☒	Antibodies
☒	☐	Eukaryotic cell lines
☒	☐	Palaeontology and archaeology
☐	☒	Animals and other organisms
☒	☐	Clinical data
☒	☐	Dual use research of concern

Methods

n/a	Involved in the study	
☒	☐	ChIP-seq
☒	☐	Flow cytometry
☒	☐	MRI-based neuroimaging

Antibodies

Antibodies used	Six antibodies were utilized. 1, Rabbit polyclonal antibody against TMEM16A (ab53212, 1:100; Abcam); 2, Goat polyclonal antibody against c-Kit (AF332, 1:20; R&D Systems); 3, Mouse monoclonal antibody against Myh11 (ab683, 1:400; Abcam); 4, Alexa Fluor 488-conjugated donkey anti-rabbit immunoglobulin G (IgG) H&L (ab150061, 1:500; Abcam); 5, Alexa Fluor 568-conjugated donkey anti-goat IgG (H+L; ab175474, 1:500; Abcam); 6, Alexa Fluor 594-conjugated donkey anti-mouse IgG (H+L; ab150112, 1:500; Abcam).
Validation	All the six antibodies have bee validated by us and other researchers.

Animals and other research organisms

Policy information about studies involving animals; ARRIVE guidelines recommended for reporting animal research, and Sex and Gender in Research

Laboratory animals	All animal procedures were approved by the Institutional Animal Care and Use Committees at the University of Massachusetts Chan Medical School (protocol number A1473) in accordance with the National Research Council Publication Guide for the Care and Use of Laboratory Animals and NIH Guide for the Care and Use of Laboratory Animal.
Wild animals	Not applicable.
Reporting on sex	Study was conducted with both sex of mice.
Field-collected samples	Not applicable.
Ethics oversight	All work with vertebrate animals was approved by by the Institutional Animal Care and Use Committees at the University of Massachusetts Chan Medical School (protocol number A1473).

Note that full information on the approval of the study protocol must also be provided in the manuscript.
